# Structural dissection of human metapneumovirus phosphoprotein using small angle x-ray scattering

**DOI:** 10.1038/s41598-017-14448-z

**Published:** 2017-11-01

**Authors:** Max Renner, Guido C. Paesen, Claire M. Grison, Sébastien Granier, Jonathan M. Grimes, Cédric Leyrat

**Affiliations:** 1 Division of Structural Biology, Wellcome Trust Centre for Human Genetics, Oxford University, Roosevelt Drive, Oxford, OX3 7BN UK; 2 Institut de Génomique Fonctionnelle, CNRS UMR-5203 INSERM U1191, University of Montpellier, Montpellier, France; 3 Science Division, Diamond Light Source Ltd., Diamond House, Harwell Science and Innovation Campus, Didcot, Oxfordshire OX11 0DE United Kingdom

## Abstract

The phosphoprotein (P) is the main and essential cofactor of the RNA polymerase (L) of non-segmented, negative‐strand RNA viruses. P positions the viral polymerase onto its nucleoprotein–RNA template and acts as a chaperone of the nucleoprotein (N), thereby preventing nonspecific encapsidation of cellular RNAs. The phosphoprotein of human metapneumovirus (HMPV) forms homotetramers composed of a stable oligomerization domain (P_core_) flanked by large intrinsically disordered regions (IDRs). Here we combined x-ray crystallography of P_core_ with small angle x-ray scattering (SAXS)-based ensemble modeling of the full-length P protein and several of its fragments to provide a structural description of P that captures its dynamic character, and highlights the presence of varyingly stable structural elements within the IDRs. We discuss the implications of the structural properties of HMPV P for the assembly and functioning of the viral transcription/replication machinery.

## Introduction

Acute respiratory tract infections constitute an important cause of mortality in children under five, with ~1.5 million fatalities reported in 2008^1^. Human metapneumovirus (HMPV), first described in the Netherlands in 2001, is a major agent of viral respiratory illness and pneumonia worldwide^2^. Although most often asymptomatic in healthy adults, HMPV can be severe in immunocompromised and elderly populations^3,4^. After the closely related respiratory syncytial virus (RSV), HMPV is recognized to be the second most important cause of viral bronchiolitis and pneumonia in young children^4,5^. It has been posited that HMPV evolved from an avian metapneumovirus-like ancestor and that there has been a zoonotic cross-species transmission event from birds to humans around 200 years ago^6^. Both HMPV and RSV are classified as members of the *Pneumoviridae*, recently elevated to family status within the viral order *Mononegavirales* ^7^. The *Mononegavirales* order harbours numerous significant human pathogens such as Ebola virus (family *Filoviridae*), measles virus (family *Paramyxoviridae*), and rabies virus (family *Rhabdoviridae*).

All mononegaviruses possess common transcription/replication strategies and a similar genome organisation^8^. In members of *Mononegavirales* the single-stranded (ss), negative-sense (−) RNA genome is protected by a sheath of oligomerised viral nucleoproteins N (NP in *Filoviridae*) which form the nucleocapsid^9–11^. This packaged form of the RNA genome prevents antiviral signalling and degradation by host nucleases, but also serves as the template for transcription and replication by the L polymerase^8,9^. The HMPV genome (~13 kB) encodes 9 genes, N-P-M-F-M2-SH-G-L, ordered from 3′- to 5′-end of the RNA which are transcribed sequentially by L^12,13^ within cytoplasmic viral inclusion bodies that serve as viral transcription/replication factories^14–16^. To transcribe viral mRNAs from the genome and to replicate progeny genomes L requires the essential ancillary factor P^17,18^. In addition, processive transcription into full-length and polycistronic mRNAs also requires the antitermination factor M2–1^19,20^. Components of the replicase/transcriptase complexes, consisting of the N, L, P, and M2-1 proteins, are important targets for the development of antiviral therapeutics^21^.

The polymerase cofactor P (VP35 in *Filoviridae*) serves as a central hub of the replicase/transcriptase by bringing together their various elements, but also performs key functions throughout the viral life cycle. In the case of HMPV, the phosphoprotein was shown to play an important role in direct cell-to-cell viral spread by co-localizing with actin and inducing membrane deformations in bronchial airway cells^22^. A role in manipulating the host immune response by preventing RIG-I-mediated sensing of HMPV viral 5′ triphosphate RNA has also been demonstrated for the phosphoprotein of the HMPV B1 strain^23^. Pneumoviral P interacts with both L and the nucleocapsid, thereby tethering the polymerase to its template^24–27^. During transcription, P also recruits the M2-1 antiterminator to the polymerase, where M2-1 is thought to bind nascent viral mRNA emerging from L^28–30^. In replication, P supplies growing progeny nucleocapsids with naive, RNA-free nucleoproteins (N^0^) for immediate, co-transcriptional packaging. This is achieved via an N-terminal region of P which binds to N^0^, thereby preventing premature RNA encapsidation and oligomerisation^31–34^. A hallmark of mononegavirus P proteins is their propensity to form distinct functional oligomers. In HMPV and RSV a central oligomerization domain facilitates the formation of P tetramers^35–37^. Polymerase cofactors from members of *Mononegavirales* feature conditionally-folded molecular recognition elements (MoREs) and extended intrinsically disordered regions (IDRs) which cannot be described by a single unique conformation in solution^26,37–41^. This characteristic seriously hampers the structural understanding of full-length P proteins by x-ray crystallography or cryo-electron microscopy and, consequently, to date these techniques have not delivered a structural description of any full-length mononegavirus polymerase cofactor. However, the use of structural information from isolated stable domains combined with small angle x-ray scattering (SAXS) and molecular dynamics simulations (MDS) can reveal an ensemble description of the entire protein. Here we have used this integrated approach to structurally dissect the functional regions of HMPV P.

## Results

### Structures of P_core_ from two new crystal forms

**Figure 1:**
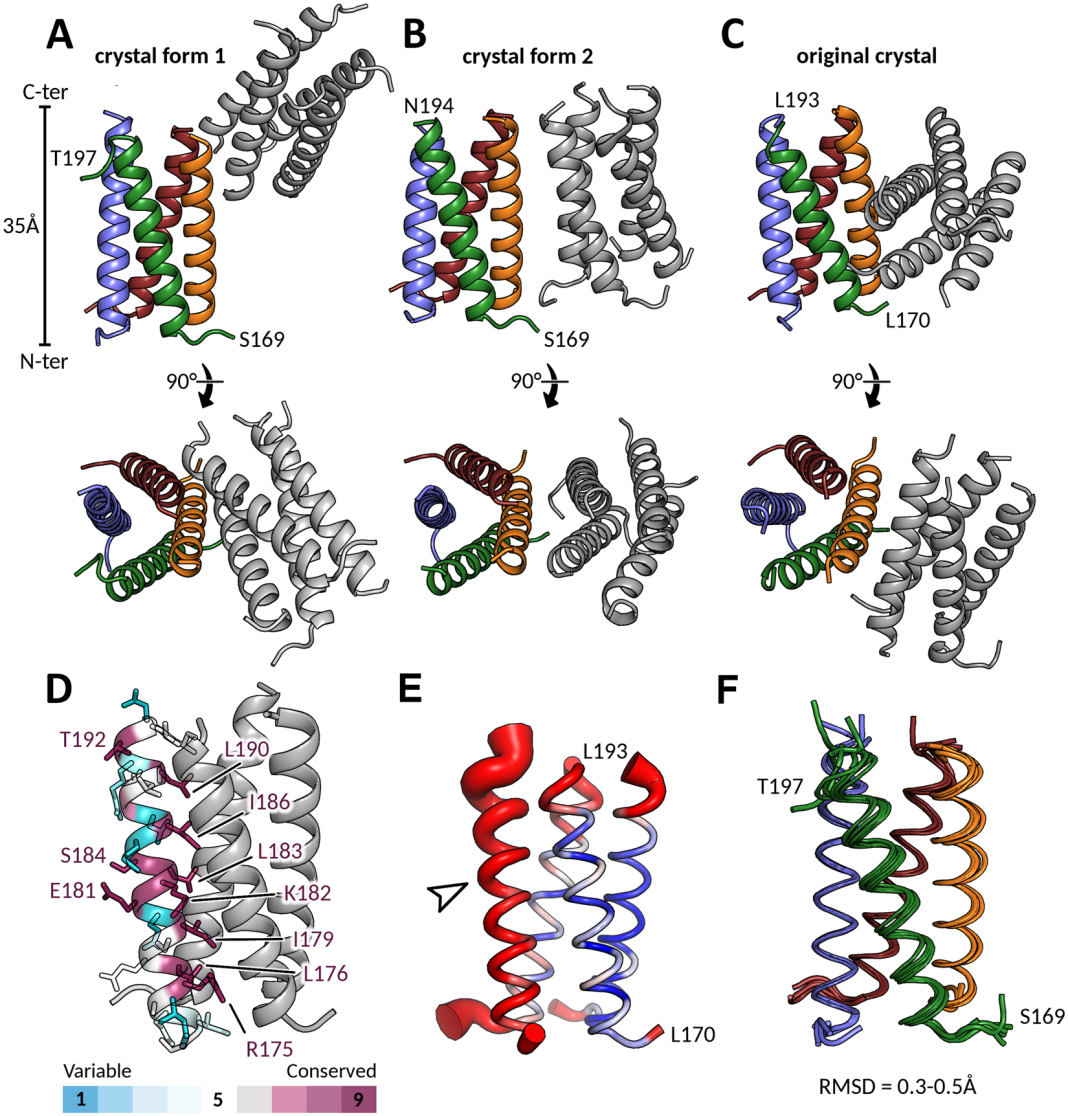
Structures of HMPV P_core_ from different crystal forms. A, B and C. Asymmetric units of two different P_core_ crystal forms shown in side view and top view orientations. In both cases the asymmetric unit contained two coiled-coil P_core_ tetramers (one depicted in grey and one coloured by chain). The structure in (**A**) is derived from the P2_1_ crystal which diffracted to 1.6 Å, while the structure in (**B**) is derived from the P2_1_2_1_2_1_ crystal at 2.2 Å resolution. The previously published structure (PDB ID: 4BXT) is shown for comparison in (**C**) highlighting the distinct packing arrangements of each crystal. (**D**) The sequence conservation within *Pneumoviridae* members was mapped onto the P_core_ structure and amino acids are coloured by conservation as indicated. For clarity, the side chains of only one of the four protomers are shown. Strictly conserved residues (coloured in deep purple) are explicitly labelled. E. B-factor putty representation of a single P_core_ tetramer from crystal form 2. Red and thick regions of the putty represent high B-factors (~50 Å^2^), whilst thin and blue regions indicate low B-factors (~20 Å^2^). A solvent accessible helix displayed significantly higher B-factors that the rest of the coiled-coil (indicated by arrow). F. Structural superposition of six P_core_ tetramers taken from crystal forms 1 and 2 and the previously published crystal (PDB ID: 4BXT).

We have previously reported the crystallographic structure of the HMPV P_core_ domain (P residues ~169–194) at an intermediate resolution of 3.1 Å^37^. Serendipitously (see Materials and Methods), we obtained two additional crystal forms of P_core_ with improved resolution, allowing us to build a higher quality model (100% Ramachandran favoured, see Table 1). The first crystal (form 1, space group P2_1_) diffracted to a resolution of 1.6 Å, while the second crystal (form 2, space group P2_1_2_1_2_1_) gave rise to diffraction data up to 2.2 Å. In all of the crystal forms, including the original crystal (PDB ID: 4BXT)^37^, the asymmetric unit is composed of two tetrameric, helical coiled-coils packing against each other in varying orientations (Fig. 1A–C). Sample electron density maps of the higher resolution P_core_ structures are shown in Supplementary Fig. S1.

**Table 1:** Crystallographic data collection and refinement statistics.

	P_core_ form 1 (PDB ID: 5OIX)	P_core_ form 2 (PDB ID: 5OIY)
**Data collection**		
Space group	P 1 21 1	P 21 21 21
Cell dimensions		
*a, b, c* (Å)	29.0, 110.4, 38.7	37.8, 45.4, 116.8
*α, β, γ* (°)	90, 95.6, 90	90, 90, 90
Resolution (Å)	36.4–1.6 (1.67–1.61)*	42.4–2.2 (2.28–2.20)
CC (1/2)	1.0 (0.5)	1.0 (0.7)
*R*_merge_	4.6 (101.4)	11.0 (275.8)
*R*_pim_	2.9 (65.3)	3.3 (81.0)
*I/σI*	13.9 (1.4)	11.9 (1.3)
Completeness (%)	99.3 (99.7)	98.7 (97.1)
Redundancy	3.3 (3.3)	12.2 (12.4)
**Refinement**		
Resolution (Å)	36.4–1.6	42.4–2.2
No. reflections	31086 (3078)	10636 (1033)
*R*_work_ */R*_free_	17.9/20.3	24.8/26.5
No. atoms		
Protein	1640	1586
Ligand	6	0
Solvent	242	67
B-factors (Å2)		
Protein	34.6	51.6
Ligand	71.1	-
Solvent	51.0	51.2
R.m.s. deviations		
Bond lengths (Å)	0.009	0.007
Bond angles (°)	1.26	1.26
Ramachandran plot quality (%)		
Favoured region	100.0	100.0
Allowed region	0	0
Outliers	0	0

^*^Highest resolution shell is shown in parenthesis.

Mapping of the sequence conservation within the *Pneumoviridae* onto P_core_ reveals the strict conservation of the hydrophobic amino acids (Leu and Ile residues) lining the interior of the coiled-coil, indicating that all family members share a similar P oligomerization region (Fig. 1D, strictly conserved residues in purple). Furthermore, Arg175 is strictly conserved in all *Pneumoviridae* and plays a role in stabilizing the quaternary structure of the tetramer by forming a salt bridge with either an Asp or Glu on the neighbouring helix. Notably, residues Ser184 and Glu181 are also invariable despite being exposed to the solvent and not directly involved in oligomerization. It is tempting to speculate that these residues may instead be involved in interactions with other viral components, for instance the L polymerase^42^.

In one of the tetramers within crystal form 2 we observed a single, solvent-exposed helix which possessed markedly blurred electron density and, as a result, significantly higher crystallographic B-factors than the three other protomers of the coiled-coil (Fig. 1E, white arrow). This suggests that, in absence of packing constraints within the crystal (as is the case for this solvent-exposed helix), the protomers of the P_core_ region are somewhat flexible and there is a certain degree of malleability of the tetramer interface. Alignment of all six tetramers from crystal forms 1 and 2 and the original crystal confirmed that there are small-scale breathing motions within the coiled-coil (Fig. 1F). This is fully consistent with the overall lower buried surface area (~4800 Å) and predicted dissociation energy (~21 kcal/mol) for HMPV P than in the more extended paramyxoviral coiled-coil oligomerization domains. For comparison, the buried surface area and predicted dissociation energy of the Nipah virus P oligomerization domain have been reported to lie between 15000–20000 Å and 140–200 kcal/mol^43^. These data indicate a flexibly associated tetramer of HMPV P protomers.

### SAXS characterization of the HMPV P constructs

**Figure 2:**
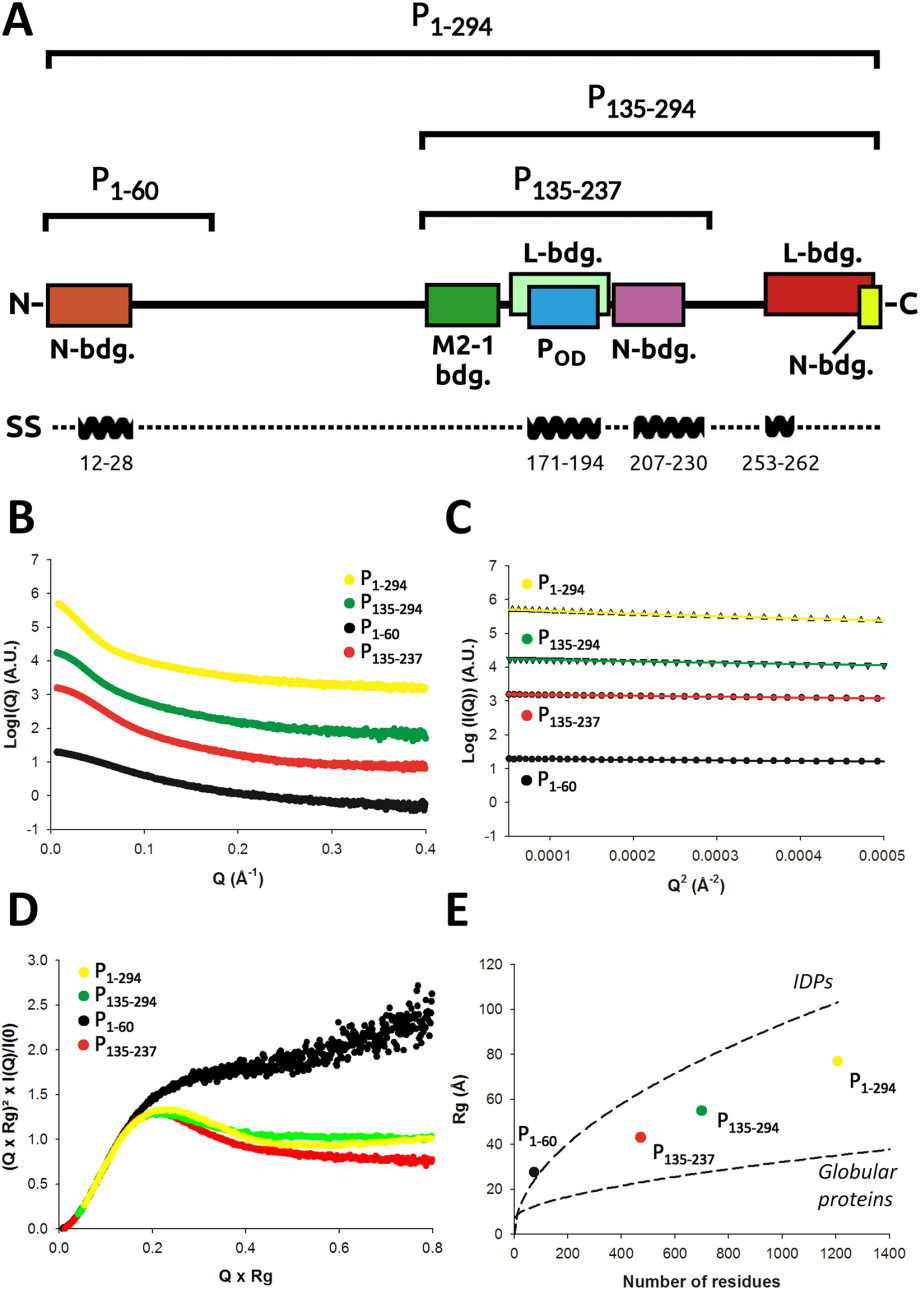
HMPV P constructs and small angle x-ray scattering experiments (SAXS). (**A**) Functional regions and HMPV P constructs used in this study. The N^0^ binding region (residues 1–28) is shown as a brown box and the crystallographically observed tetramerization domain P_core_ in blue. The putative M2-1, L and N-RNA binding regions are indicated based on homology with human/bovine respiratory syncytial viruses (RSV)^16,26,27,42,60,66,99,100^. The location of predicted α-helices is indicated on a separate bar below. (**B**) Measured SAXS profiles of P_1–60_ (black), P_135–237_ (red), P_135–294_ (green) and P_1–294_ (yellow). (**C**) Corresponding Guinier plots, showing linear behavior in the low q range. (**D**) Normalized Kratky plots. (**E**) The radius of gyration versus number of residues for the different HMPV constructs is shown as colored spheres, and is compared with empirical laws for globular^45^ and intrinsically disordered proteins (IDPs)^46^.

Next, we turned to SAXS in order to obtain structural information in the solution state for the different functional regions of P (Fig. 2). In total 4 P constructs were characterized by SAXS: 1) the N-terminal region (P_1–60_) which encompasses the N^0^ binding site, 2) the central region (P_135–237_) composed of the M2-1 binding site, the tetramerization domain P_core_ and a putative nucleoprotein binding region, 3) the P_135–294_ construct, which includes potential L and N binding regions at its C-terminus, and 4) the full-length P protein (P_1–294_) (domain annotations are shown in Fig. 2A and Supplementary Fig. S2). Our approach of splitting the protein into different fragments enables more accurate X-ray scattering profiles to be obtained compared to focussing on the full-length protein only, thus increasing the overall structural information content that can be extracted from the SAXS data. For each construct, high-quality SAXS profiles could be obtained (Fig. 2B), and samples were free from aggregates as evidenced by the linearity of the Guinier region (Fig. 2C). Parameters extracted from the SAXS data are summarized in Table 2. Molecular weights were estimated based on calculation of the concentration-independent volume of correlation V_C_, as defined in^44^, and were found to be consistent with the theoretical molecular weight expected for each construct. In the case of the full-length P, a SEC-MALLS profile was also recorded, which confirmed the sample monodispersity, oligomerization state, and molecular weight (Supplementary Fig. S3, MW_MALLS_ = 125 kDa ± 12 kDa and MW_theor._ = 134.8 kDa). The SAXS-derived radii of gyration (R_g_) were independent of concentration for P_1–60_ and P_135–237_. On the contrary P_135–294_ and P_1–294_, respectively, were observed to significantly compact or expand with increasing protein concentration indicating the presence of a structure factor contribution to the data, which was treated through data merging of different protein concentrations (Table 2).

**Table 2:** SAXS-derived parameters.

HMPV P construct	Buffer conditions	c (mg/ml)	MW (kDa)	R_g_ (nm)	χ_exp_	Optimizedensemble size**
P_1–60_	Buffer A*	4	11	2.82 ± 0.04	0.693	6
P_1–60_	Buffer A	2	9	2.78 ± 0.03	0.631	9
P_1–60_	Buffer A	1.5	10	2.75 ± 0.04	0.628	10
P_1–60_	Buffer A	1	10	2.74 ± 0.05	0.616	12
P_135–237_	Buffer A	2	55	4.26 ± 0.05	0.608	7
P_135–237_	Buffer A	4	54	4.29 ± 0.03	0.656	7
P_135–237_	Buffer A	8	57	4.27 ± 0.01	1.094	5
P_135–237_	Buffer A + 1M Gdn-HCl	4	50	4.76 ± 0.06	0.875	5
P_135–237_	Buffer A + 2M Gdn-HCl	4	54	5.22 ± 0.10	0.860	3
P_135–237_	Buffer A + 3M Gdn-HCl	4	57	5.25 ± 0.13	0.742	5
P_135–294_	Buffer A	1	93	5.66 ± 0.11	N.D.	N.D.
P_135–294_	Buffer A	1.5	82	5.63 ± 0.11	N.D.	N.D.
P_135–294_	Buffer A	2	81	5.65 ± 0.03	N.D.	N.D.
P_135–294_	Buffer A	3	81	5.36 ± 0.02	N.D.	N.D.
P_135–294_	Buffer A	merged	83	5.68 ± 0.03	0.506	6
P_1–294_	Buffer A	0.3	122	7.13 ± 0.08	N.D.	N.D.
P_1–294_	Buffer A	0.4	111	7.19 ± 0.08	N.D.	N.D.
P_1–294_	Buffer A	0.9	130	7.32 ± 0.08	N.D.	N.D.
P_1–294_	Buffer A	1.1	137	7.45 ± 0.05	N.D.	N.D.
P_1–294_	Buffer A	1.5	112	7.49 ± 0.06	N.D.	N.D.
P_1–294_	Buffer A	2.0	141	7.53 ± 0.06	N.D.	N.D.
P_1–294_	Buffer A	merged	128	7.25 ± 0.08	1.738	4

*Buffer A: 20 mM Tris pH 7.5 150 mM NaCl.

**Optimized ensemble size: The optimum selected ensemble size and relative weights of the models were determined automatically by GAJOE using default parameters.

We used normalized Kratky plots to obtain a semi-quantitative view of the level of intrinsic disorder present in each construct. As can be seen in Fig. 2D, all constructs showed a mixed profile indicating a balance between order and disorder, with the exception of P_1–60_ which displayed a typical intrinsically disordered protein (IDP) Kratky plot. This is likely due to the absence of the tetramerization domain, the most ordered region in P. A similar behaviour was observed when the experimental R_g_ of each construct was placed on a R_g_ versus number of residues plot and compared with the empirical laws available for both globular^45^ and intrinsically disordered proteins^46^ (Fig. 2E). P_1–60_ appeared as a canonical IDP while the tetrameric constructs showed an intermediate behavior between fully folded proteins and IDPs. Of note, the full-length P_1–294_ was slightly offset towards the IDP curve compared to P_135–237_ and P_135–294_, consistent with an increase in intrinsic disorder upon inclusion of the N-terminal region. We then used computational modeling in combination with ensemble optimization to derive SAXS-validated ensembles of atomic models for each construct.

### The N-terminal region of P shows α-helical propensity and is in equilibrium between extended and more compact conformers

**Figure 3:**
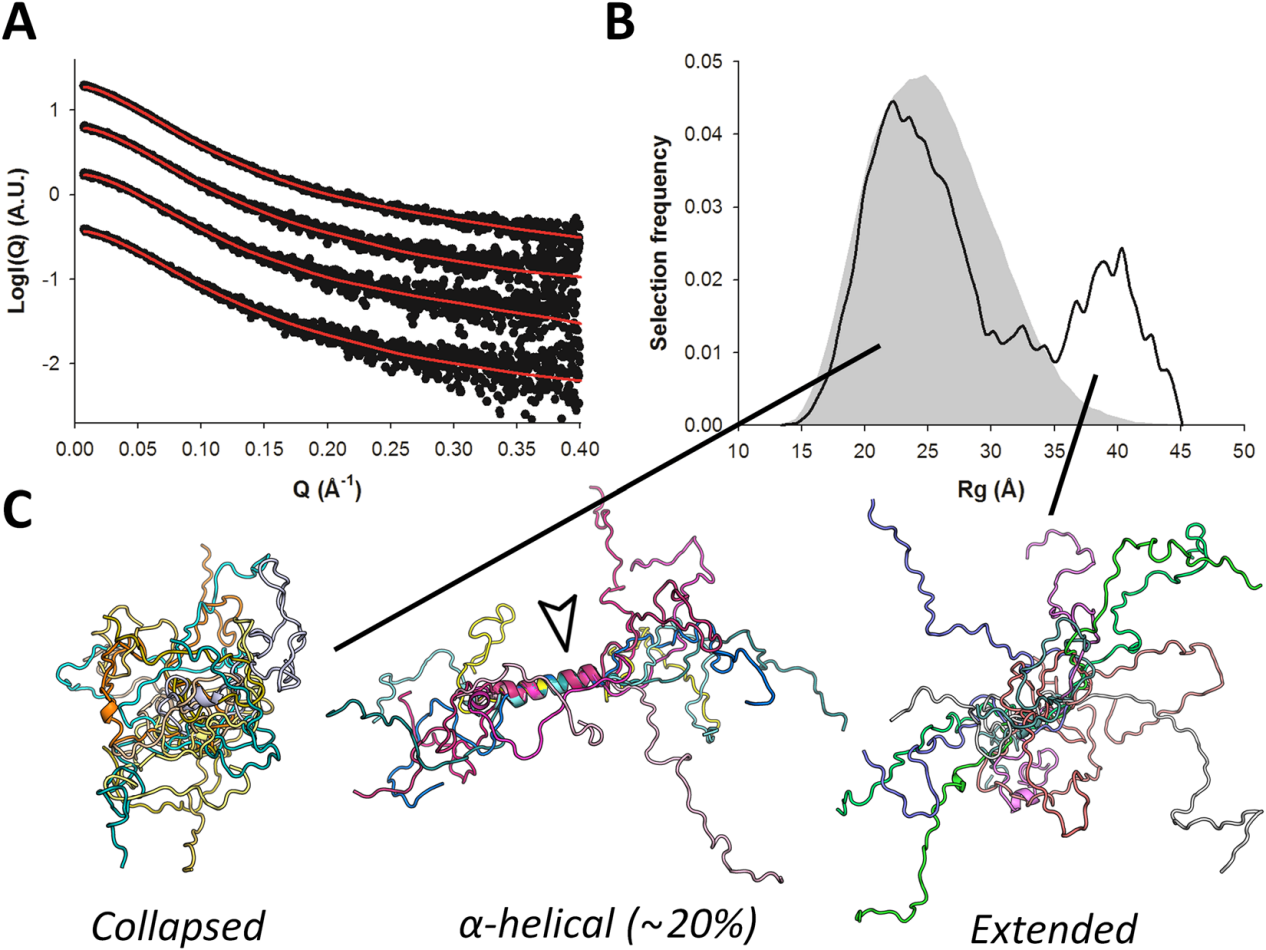
SAXS-based ensemble analysis of P_1–60_. (**A**) Fitted SAXS profiles of P_1–60_ for 4 different concentrations. Experimental scattering curves are shown as black spheres with optimized ensembles (OEs) fits shown as red lines. The curves are ordered by increasing protein concentrations (Table 2) from bottom to top. (**B**) Radius of gyration distributions for the initial pool ensemble (gray area) and for the OEs (black line), averaged over all protein concentrations. (**C**) Families of models extracted from the OEs were classified into “collapsed” or “extended” based on their radius of gyration. About 20% of models with α-helical structure are also present in the OEs.

All-atom models of P_1–60_ were generated using the state-of-the-art program flexible-meccano^47^ and used for ensemble optimization of the available experimental SAXS curves, resulting in a high-quality fit to the data (χ_exp_ ~ 0.6 - Table 2, Fig. 3A). Because no significant concentration dependence of the R_g_ was observed, an average R_g_ distribution was calculated and is shown in Fig. 3B, revealing an equilibrium between two populations of different sizes centered around 2.2 nm and 4.0 nm. Representative models of these two populations are shown in Fig. 3C. A significant fraction of the selected models (~20%) displayed α-helical structure in the previously reported N^0^ binding region (Fig. 3C) indicating that this MoRE is able to partially fold as an α-helix in the absence of its binding partner.

### Ensemble analysis of the central region of P indicates the presence of loose tertiary structure located C-terminally of P_core_

**Figure 4:**
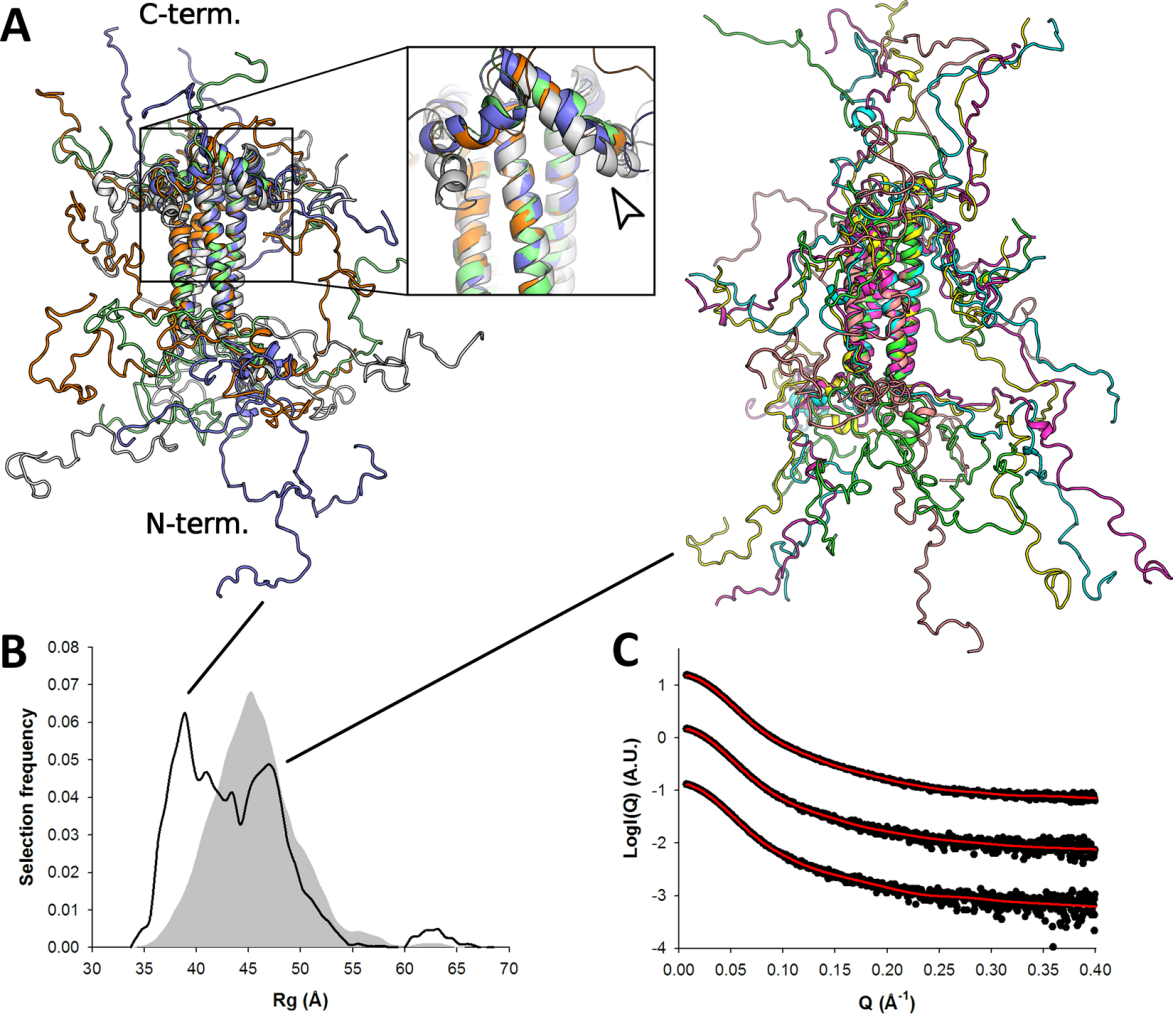
SAXS-based ensemble analysis of P_135–237_. (**A**) Models extracted from the OEs were categorized based on the presence (left) or absence (right) of C-terminal α-helical elements (residues 202–219) packed against the coiled coil region. A zoom of the C-terminal α-helical elements is shown in inset and highlighted by an arrow. (**B**) Radius of gyration distributions for the initial pool ensemble (gray area) and for the OEs (black line), averaged over all protein concentrations. (**C**) Fitted SAXS profiles of P_135–237_. Experimental scattering curves are shown as black spheres with optimized ensembles (OEs) fits shown as red lines. The three curves are ordered by increasing protein concentrations (Table 2) from bottom to top.

Ensembles of atomic models of P_135–237_ were generated by combining classical and coarse-grained atomistic molecular dynamics simulations and using the available SAXS-validated models of P_158–237_ as templates (see material and methods)^37^. The two constructs differ only by the presence of the putative M2–1 binding region approximately located between residues 135 and 158, which mainly adopts extended conformations within the selected ensembles, although residual α-helical structure between residues 138–145 is present in a small fraction of the models. The ensemble optimization results show that the selected ensembles (Fig. 4A) successfully reproduced the experimental data (0.6 < χ_exp_ < 1.1 - Table 2, Fig. 4C). The R_g_ distribution highlights the equilibrium between two main populations (Fig. 4B) which differ in the conformation of their C-terminal regions (Fig. 4A, inset). Residues 204–219 (which overlap with a recently proposed secondary N-binding site in RSV^26^) tend to form an α-helix (α_204–219_) that packs against the P_core_ mainly through hydrophobic interactions. This relatively unstable structural motif is present in about 30% of the selected models and gives rise to a somewhat more compact population, shifting down the R_g_ values by about 1 nm compared to the other, more extended population. Interestingly, these ordered conformations of residues 204–219 have originally been observed when modeling P_158–237_ and were found to be relatively stable for several hundreds of nanoseconds of classical MDS both in our previous work^37^ and in this study (not shown). It is worth noting that when performing the ensemble optimization using a pool in which the compact population of models was excluded, we could observe a significant deterioration of the χ_exp_ value for the highest concentration SAXS curve from 1.09 to 1.49, indicating that the presence of a packed α_204–219_ in the ensemble models is important to correctly reproduce the experimental SAXS profile.

**Figure 5:**
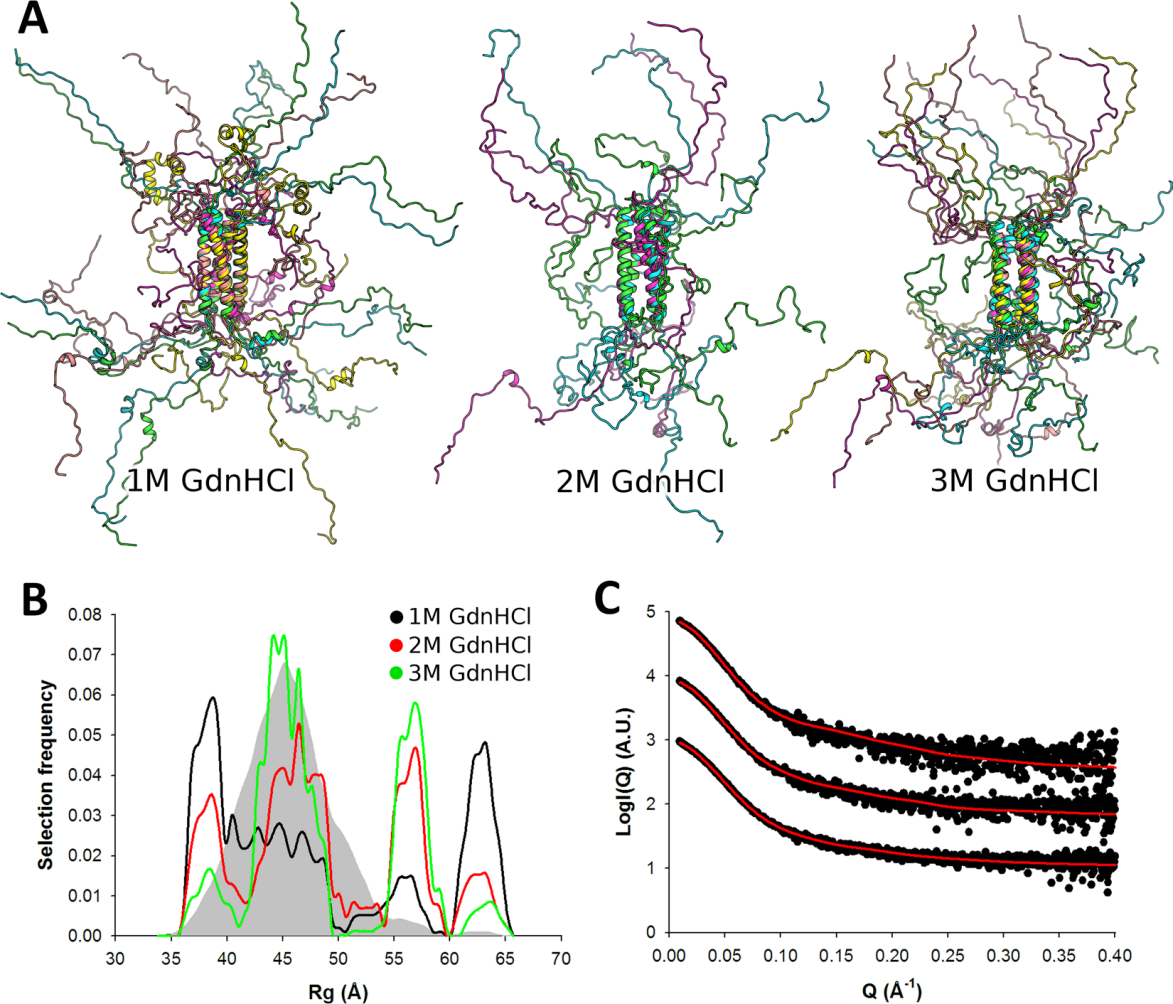
Effect of guanidinium hydrochloride on the solution structure of P_135–237_. (**A**) Models from the OEs obtained in the presence of increasing concentrations of guanidinium hydrochloride (Gdn-HCl) showing the progressive disappearance of the α-helical elements outside of P_core_. (**B**) Radius of gyration distributions for the initial pool ensemble (gray area) and for the OEs obtained in the presence of 1 M Gdn HCl (black line), 2 M Gdn HCl (red line) and 3 M Gdn HCl (green line). (**C**) Fitted SAXS profiles of P_135–237_ measured in the presence of increasing concentrations of guanidinium hydrochloride (1, 2 and 3 M, from bottom to top). Experimental scattering curves are shown as black spheres with optimized ensembles (OEs) fits shown as red lines.

To further assess the validity of our ensemble analysis procedure, SAXS profiles of P_135–237_ were recorded at increasing concentrations of guanidinium hydrochloride (Gdn-HCl) (Fig. 5 and Supplementary Fig. S4). Addition of 1, 2 and 3 M Gdn-HCl resulted in the progressive disappearance of the most compact population and a concomitant increase in the proportion of extended models (Fig. 5A and B). As can be seen in Fig. 5A, the population of models where α_204–219_ packs against the neighboring P_core_ is completely absent in the 1 M Gdn HCl selected ensemble, while residual α-helical structure in this region is retained. At higher Gdn-HCl concentrations, these secondary structure elements are also lost. Interestingly, P_core_ was found to remain stable at all Gdn-HCl concentrations indicating a highly stable tetrameric core.

### The C-terminal region of P may stabilize the loose tertiary structure present in the central region

**Figure 6:**
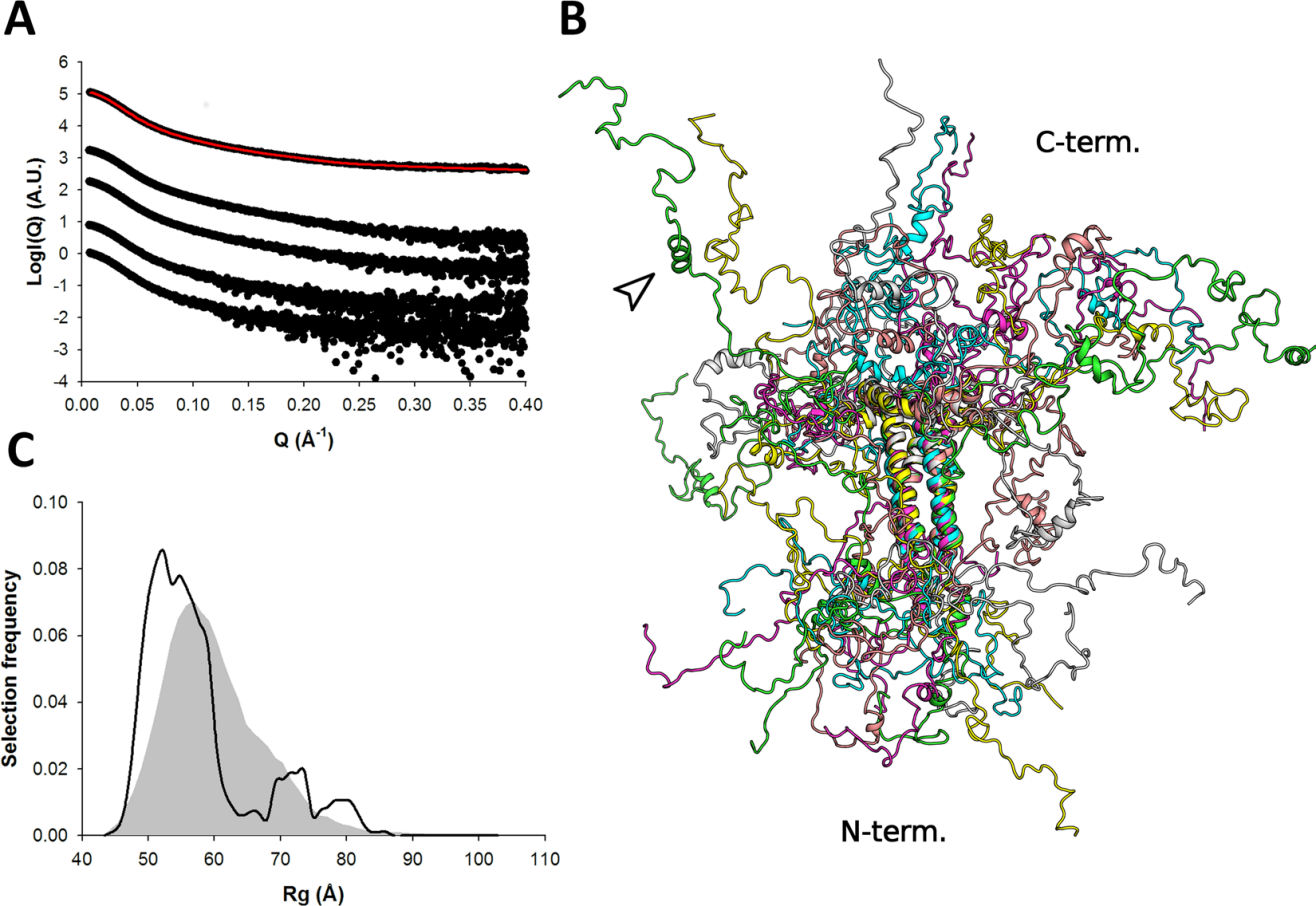
SAXS-based ensemble analysis of P_135–294_. (**A**) SAXS profiles of P_135–294_. Experimental scattering curves are shown as black spheres with the fit to the optimized ensemble (OE) shown as a red line for the merged data curve. The curves are ordered by increasing protein concentrations (Table 2) from bottom to top. The top curve corresponds to a merging of high and low concentrations to remove structure factor contributions. (**B**) Models extracted from the OE obtained using the merged SAXS profile. The short α-helical motif near the C-terminus (residues 253–262) is indicated by an arrow. (**C**) Radius of gyration distributions for the initial pool ensemble (gray area) and for the OE (black line).

Models of P_135–294_ were obtained using coarse-grained atomistic MDS based on the P_135–237_ modeling results. Again, the quality of the fit to the experimental data was very good (**χ**_**exp**_ = 0.5 - Table 2, Fig. 6A). The R_g_ distribution is dominated by a main population centered around 5–6 nm in equilibrium with more extended models (Fig. 6C). At high protein concentrations, we observed a significant decrease of the measured R_g_ from 5.66 to 5.36 nm (Table 2). This concentration-dependent R_g_ decrease might be linked to the presence of a highly negatively charged patch present in the C-terminal part of the molecule (Supplementary Fig. S5), leading to long range intermolecular repulsion. About 55% of selected models appear to have the packed α_204–219_, suggesting that the C-terminal region of the protein might stabilize the cap of helices located C-terminally to the tetramerization domain (and also observed with the P_135–237_ construct). The 75 C-terminal amino acids adopt various disordered conformations, with a significant fraction of the selected models displaying a short α-helical motif near their C-terminus in the predicted region (residues 253–262, at the beginning of the putative L-binding site) (Fig. 6B). It is however not possible to ascertain the presence of this motif based on SAXS data alone for such a large construct, as only a modest fraction of the scattering arises from this region of the molecule.

### Ensemble structure of the full-length phosphoprotein

**Figure 7:**
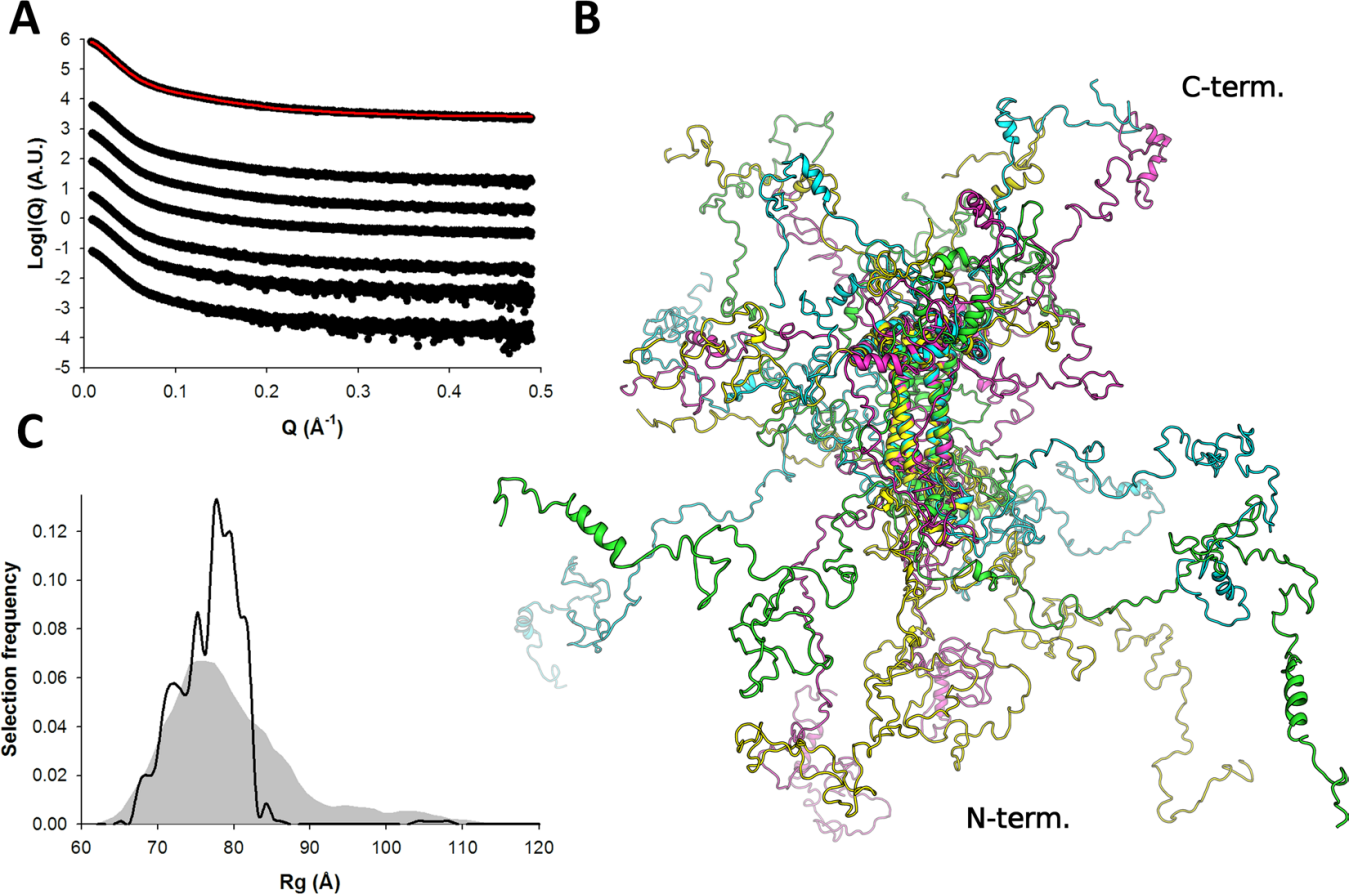
SAXS-based ensemble analysis of P_1–294_. SAXS profiles of P_1–294_. Experimental scattering curves are shown as black spheres with the fit of the optimized ensemble (OE) shown as a red line. The curves are ordered by increasing protein concentrations (Table 2) from bottom to top. The top curve corresponds to a merging of high and low concentrations to remove structure factor contributions. (**B**) Radius of gyration distributions for the initial pool ensemble (gray area) and for the OE (black line). (**C**) Models extracted from the OE obtained by fitting the merged data curve.

Finally, we generated an ensemble of atomic models of P_1–294_ using coarse-grained MDS based on the results of the P_60_ and P_135–294_ modeling and performed ensemble optimization against the experimental SAXS data. We were able to adequately fit the data with χ_exp_ = 1.7 (Fig. 7A and Table 2). A representative ensemble of models optimized using the merged SAXS curve is shown in Fig. 7C. The full-length phosphoprotein appears as a large, tentacular molecule with maximal intramolecular distances (D_max_) roughly comprised between 25 and 35 nm, and a partitioning of the N-terminal and C-terminal intrinsically disordered extensions on each side of P_core_. The R_g_ distribution shows a main population centered around 7–8 nm (Fig. 7B) and about 60% of selected models displayed a packed α_204–219_. Contrary to P_135–294_, higher protein concentrations lead to higher apparent R_g_ values that increase from 7.1 to 7.5 nm in the (relatively narrow) concentration range used (Table 2). This concentration-dependent increase in R_g_ indicates attractive interparticle interference which possibly results from the presence of both highly negatively and positively charged patches located in the N-terminal and C-terminal regions (Supplementary Fig. S5).

## Discussion

The essential polymerase cofactor P from *Pneumoviridae* is a multifunctional hub which interacts with numerous components of the viral RNA-synthesis machinery. It also possesses large IDRs and can therefore not easily be characterized by classical structural biology methods, such as X-ray crystallography. NMR studies of the RSV P protein have shown that IDRs account for around 80% of the protein^26^. Analysis of whole eukaryotic genomes has suggested that as much as 41% of protein sequences contain IDRs of significant length^48^ and research on IDPs indicates that these regions are frequently involved in protein-protein interactions (reviewed in^49^). *Pneumoviridae* P is commonly described as possessing an N-terminal IDR (mainly associated with binding RNA-free N) and a C-terminal IDR (mainly associated with binding N-RNA), which are separated by a central tetramerization module, P_core_^24,32,37,50^. In this study we present a description of the structure and dynamics of HMPV P, using a combination of crystallography of stable/bound domains and SAXS ensemble analysis. We show that full-length phosphoprotein exists in solution as an ensemble of inter-converting conformers with a tentacular architecture. The four long N-terminal IDRs and the four somewhat shorter C-terminal IDRs are partitioned by the central tetramerization domain owing to the parallel orientation of P_core_ α-helices. The P protein tetramer (1176 residues) has huge dimensions, with an average R_g_ of 7.5 nm and an average D_max_ of about 30 nm. For comparison, the entire L polymerase (2109 residues) of vesicular stomatitis virus (VSV), which is likely similar in size as HMPV L polymerase, displays a R_g_ of 3.9 nm and a D_max_ of about 13 nm, based on its cryo-electron microscopy structure^51^. Undoubtedly, the large dimensions and flexibility of the P protein reflect its functions in viral transcription and replication which require the simultaneous binding and adequate positioning of the N^0^, the M2-1 anti-terminator, the L polymerase and the nucleocapsid. In addition, simultaneous binding of P to multiple N-RNA subunits may influence the conformation and curvature of the nucleocapsid.

### Conformation of the N-terminal region

The disordered N-terminal region of P has been implicated in chaperoning the N protein, keeping it in an RNA-free state prior to nucleocapsid assembly, for a range of negative strand viruses^32,33,39,52,53^. This is important as N may otherwise unproductively bind to host nucleic acids. N-chaperoning is facilitated by a MoRE located towards the very N-terminus of the P-protein, which folds upon binding nascent and RNA-free nucleoproteins^31,39^. Kratky analysis of the first 60 N-terminal residues of HMPV P (P_60_) confirmed the disordered character of this region. However, in our ensemble analysis 20% of the models exhibited helical secondary structure within residues 13–28, corresponding to the same helix that is observed in the crystal structure of HMPV P bound to N_0_^32^. Interestingly, a recent NMR study similarly observed a 20% helical propensity in the equivalent residues (amino acids 12–24) of RSV P^26^. Such pre-formed secondary structure elements in the *apo* form have been suggested to lead to increased association rates to the binding partner via conformational selection^49^. Rapid on-rates may be especially important in the case of N-chaperoning as N^0^ needs to be captured by P before unspecific RNA-uptake and polymerization can take place, leading to a dead-end state for N. Following initial binding, the pre-formed helix may act as a nucleus from which the N-P binding interface is extended to what is observed in the crystallographic structure (encompassing P residues 1–28) via a dock-and-coalesce mechanism^54^.

### Conformation of the M2-1 binding region

Pneumoviral P proteins recruit the additional processivity factor M2-1 to the viral transcription machinery, forming an extended non-globular complex^28,29,55^. HMPV M2-1 is a highly dynamic, tetrameric, RNA-binding protein featuring a CCCH-type zinc-finger on each protomer, which is involved in recognition of nucleic acids^30,56^. The M2-1 protein is thought to bind to nascent RNA emerging from the viral polymerase, thus inhibiting premature termination of transcripts via an as-of-yet undetermined mechanism^19,20,57–59^. Mutational studies have mapped RSV P residues 100–120 as the M2-1 interaction site^60^, which roughly corresponds to the region around residues 135–158 in HMPV. In contrast to the significant fraction of pre-formed N^0^-binding region described above, our ensembles display only marginal residual secondary structure within the M2-1 binding site of *apo* P. In-line with this observation, the NMR study of RSV P could detect a small segment of five residues (RSV amino acids 98–103) with only 8% helical propensity at the M2-1 binding region^26^. In this case, recruitment of M2-1 by P may involve folding-upon-binding of an otherwise highly unstructured region. M2-1 and P may thus form a so-called fuzzy complex, in which substantial flexibility is retained even within the bound form^61^.

### Conformation of the C-terminal IDR

Previous crystallographic and solution scattering studies of Nipah virus^43,62^ and Sendai virus P^63^ have highlighted a cap-like structure of helices folding back onto the N-terminal part of the oligomerization domain. However, the cap was not observed in the crystal structures of the helical coiled-coil oligomerization domains of Measles virus P^64^, Mumps virus P^65^, or HMPV P^37^. Although not present in the crystal, in our HMPV P solution ensembles we find a compact subpopulation of conformers which consistently features a cap of helices (amino acids 204–219) located C-terminally of the tetramerization domain. It is quite possible that the cap is a specific feature of pneumoviral P proteins that exists in solution but is insufficiently stable, at least in HMPV, to be observed in the crystalline state. Deletion studies for bovine respiratory syncytial virus (BRSV) P have suggested that the region corresponding to the cap in HMPV may represent a secondary nucleoprotein binding site, besides the primary one at the very C-terminus of P^66^. In addition, a recent NMR study of RSV P has identified a helical region partially overlapping with the corresponding cap-forming residues in HMPV^26^. The authors observed line broadening in this helix upon adding the N-terminal domain of the nucleoprotein, strongly indicating that these do indeed interact. It is worth noting that the directly adjacent oligomerization region has been suggested to interact with the large L polymerase, at least in BRSV^42^. The secondary N-binding site of the cap-helices may thus bring L and N into close vicinity and may facilitate the interplay of N with L during the viral RNA-synthesis cycle. This is in line with mutational studies in RSV which have revealed that mutation of a conserved charged cluster within the cap to alanines (corresponding HMPV residues R215/E216/E217) abrogated all reporter gene activity in a minigenome assay^67^. The R215/E216/E217 cluster of the cap-structure in HMPV P is solvent accessible in our ensembles and may thus be positioned ideally to interact with further viral components.

Our structural ensembles of P_135–294_ and P_1–294_ feature a C-terminal α-helix in the predicted region (residues 253–262), which is part of a putative L-binding site that was mapped by mutagenesis studies in HRSV^27^. Although it is difficult to validate the presence of this secondary structure element on the basis of SAXS data alone due to the weak contribution to the scattering signal arising from this region of the molecule (within the experimental SAXS profiles), it is worth mentioning that this region was suggested to fold upon binding to the L polymerase based on the effect of point mutations on HRSV RNA synthesis^27^. However, this region of the P protein is poorly conserved between RSV and HMPV owing to the insertion of a highly acidic stretch of about 18 residues in the middle of the L binding site in the HMPV P sequence (Supplementary Fig. S2), and no residual α-helical structure could be detected in the equivalent region of RSV P by NMR^26^.

### Long range effects modulating P conformational ensembles

The structural characterization of P_135–294_ and P_1–294_ also revealed the ability of specific IDRs of the P protein to influence the conformation of other parts of the protein. In particular it was observed that a significantly higher proportion of models featuring the α_204–219_ cap-like structure is present in P_135–294_ and P_1–294_ structural ensembles compared to P_135–237_ (55–60% versus 30%). The ability of the C-terminal region of P to stabilize this α-helical structure might explain its absence from the crystal structures of P_core_ (as the P constructs used for crystallization required C-terminal degradation in order to yield diffracting crystals, which in turn destabilizes the cap-like structure). We hypothesize that this property might arise from the highly acidic nature of the C-terminal region of P which contains a large number of negatively charged residues arranged in repetitive blocks (for example residues 140–170 and residues 263–294, Supplementary Figs S2 and S5), leading to intramolecular electrostatic repulsion. Interestingly, the full-length P protein SAXS profiles display higher apparent R_g_ values at high protein concentrations, indicating attractive interparticle interference. Based on the distribution of charged residues along the P sequence, it is tempting to speculate that the presence of basic patches of residues within the N-terminal IDR (Supplementary Fig. S5) combined with the acidic C-terminal IDR is responsible for this attraction.

### Possible implications for viral inclusion bodies formation

The presence of a structure factor contribution in the SAXS profiles of P within the relatively small concentration range used for measurements (0.3 to 2 mg/ml) shows that the protein has a propensity to affect the structure of the liquid itself when present at high concentrations. This property might be relevant to viral replication in physiological conditions as pneumoviral replication was shown to occur in segregated cytoplasmic inclusion bodies which harbour the components of the replication machinery and concentrate viral proteins^68,69^. Furthermore, expression of N and P was necessary and sufficient to induce the formation of cytoplasmic inclusions in HMPV and HRSV^15,16,70^, but also in more distantly related viruses such as human parainfluenza virus type 3^71^ or rabies virus^72,73^. Recently, there is great interest in the ability of proteins with IDRs to form phase-separated micro-compartments without the need of lipid bilayers, especially in conjunction with molecular crowding^74,75^ (cellular examples of such membrane-less compartments are Cajal bodies or cytoplasmic stress granules). These phase-separated organelles feature higher local concentrations of a subset of factors which are often relevant for a functionally related pathway. Typically, proteins with low-complexity regions, low sequence diversity, multivalent binding properties, and blocks of oppositely-charged residues are potentially able to form these biomolecular condensates under specific conditions. The amino-acid compositions of many mononegavirus P proteins satisfy these prerequisites, with HMPV P possessing low-complexity regions, and being composed of over 35% Lys, Arg, Glu, and Asp, often arranged in repetitive blocks. Because each N molecule displays 2 P binding sites, it is tempting to speculate that binding of P proteins to N-RNA and N^0^ in cells would result in very high local concentrations of P. This, in turn might facilitate the formation of phase-separated microenvironments, constituting highly specialized viral RNA-synthesis and replication micro-factories. Phosphorylation of constituent proteins has previously been shown to control phase-separating behaviour^76,77^. Similarly, phosphorylation of P-proteins might modulate their general phase-separating behaviour, instead of or along with specific protein-protein interactions, thus controlling inclusion body formation.

In summary, we have shown that HMPV P is a highly dynamic protein that is composed of a stable helical tetramerization domain flanked by large N-terminal and C-terminal IDRs that sample a large volume in solution and display varying degrees of preformed structural elements. We found that the N-terminal N^0^ binding site contained a significant proportion of α-helical structure and that the region C-terminally adjacent to the tetramerization domain had a tendency to form a cap-like helical structure that mapped to a putative N- RNA binding site. Our results further suggested that this cap-like structure might be stabilized by the presence of the full C-terminal IDR, and that the full-length phosphoprotein’s basic and acidic patches of residues may play a role in viral inclusion body formation by inducing long range intermolecular attraction and facilitating the formation of phase-separated microenvironments.

## Material and Methods

### Protein cloning, expression and purification

The regions of the HMPV P gene (strain NL1-00, A1, GenBank: AAK62966.1) encompassing phosphoprotein residues 1–60, 135–237, 135–294, and 1–294 were amplified by polymerase chain reaction and cloned into pOPINF (P 1–60, 135–237 and 135–294) or pOPINE (P1-294) plasmids^78^ using the In-Fusion system (TAKARA CLONTECH) following the instructions of the manufacturer. Recombinant proteins expressed from pOPINF feature a N-terminal (His)_6_-tag followed by a 3C protease site, while pOPINE has a C-terminal (His)_6_-tag. All constructs were verified by nucleotide sequencing.

The (His)_6_-tagged constructs were transformed into Rosetta2 *E. coli* cells for recombinant expression. *E. coli* were grown at 37 °C in terrific broth (TB) in presence of appropriate antibiotics to an OD_600_ of ~0.8 and expression was induced by addition of 1 mM β-D-1-thiogalactopyranoside (IPTG). Following induction, the cells were incubated at 18 °C overnight while shaking and were subsequently harvested by centrifugation (18 °C, 20 min, 4000×g). Cell pellets were resuspended in 20 mM Tris, pH 7.5, 500 mM NaCl and then lysed by sonication. The lysate was centrifuged for 45 min at 4 °C and 50000×g. The cleared lysate was syringe-filtered (0.45 μm pore size, MILLIPORE) and transferred onto a column packed with pre-equilibrated Ni^2+^-NTA Agarose (QIAGEN). Following several washes, the (His)_6_-tagged samples were eluted from the beads with 20 mM Tris, pH 7.5, 150 mM NaCl, 300 mM imidazole. Finally, the samples were subjected to size exclusion chromatography and buffer exchanged into 20 mM Tris, pH 7.5, 150 mM NaCl. For small-angle X-ray scattering experiments, proteins were concentrated on-site with centrifugal filter units (MILLIPORE).

### Small angle X-ray scattering experiments

SAXS measurements were performed on beamline BM29 (P_1–60_, P_135–237_ and P_135–294_) and former beamline ID14-3 (P_1–294_) at the European Synchrotron Radiation Facility (ESRF), Grenoble, France. Samples were kept at 20 °C and data were collected at a wavelength of 0.0995 nm and a sample-to-detector distance of 1 m. 1D scattering profiles were generated and buffer subtraction was carried out by the automated data processing pipeline available at BM29 (ID14-3). The radius of gyration was determined with the program PRIMUS^79^ according to the Guinier approximation at low *Q* values, and molecular weights were estimated based on^44^. In the case of P_135–294_ and P_1–294_, which displayed concentration dependent changes in the SAXS profile at low *Q* values, a merged curve was produced by combining the low *Q* region of data measured at low concentration with the high *Q* region of the high concentration SAXS profiles. These particle interference-free merged curves were used for subsequent analysis.

### Structural modelling and molecular dynamics simulations

Different strategies were used to obtain all atom models for each construct. 1000 models of the backbone of monomeric P_1–60_ were obtained using the program flexible-meccano^47^, with an α-helical propensity of 50% for residues 14–26 which were observed to form an α-helix when complexed with the HMPV N protein^32^. Protein side chains were then added using the program SCCOMP^80^.

Initial models of P_135–237_ were built based on three families of models extracted from our previous HMPV P modeling study that were found to accurately reproduce SAXS data for this P_158–237_^37^. All three models are composed of a tetrameric coiled coil ranging from residues 168 to 198, with disordered residues at the N-terminus that have residual α-helical structure. In the first model, residues 202–219 adopt α-helical structures that pack laterally against the C-terminal part of the coiled coil region, while residues 220–237 are extended. In the second model, residues 208–237 form an α-helix consistently with secondary structure predictions, and in the third model, residues 168–237 are in an extended conformation with no secondary or tertiary structure. The region composed of residues 135–158 (including the N-terminal His6-3C site) was obtained based on a LOMETS model^81^ adopting a relatively extended conformation which was grafted onto the three initial models of P_158–237_. Two additional starting models were generated by shortening the coiled coil by one helix turn at its C-terminus in the third model (to match the x-ray structure) and by further removing residual helical structure in the N-terminal region (that had been carried out from previous classical M.D. simulations). All five starting models of P_158–237_ were then simulated in GROMACS^82^ using either an atomistic coarse-grained structure-based model (SBM)^83,84^, or explicit solvent classical molecular dynamics simulations (MDS).

In the case of the SBM MDS, a timestep of 0.0005 time units was used and the simulation was coupled to a temperature bath via Langevin dynamics. A single 100 ns trajectory was obtained for each starting model, and snapshots were extracted every 50 ps leading to an ensemble of 5000 models. In the case of classical MDS, we generated multiple trajectories for an aggregated simulation time of ~660 ns. MDS was performed using the amber99SBws forcefield^85^ which has been developed to reproduce the properties of intrinsically disordered proteins. At the beginning of each simulation, the protein was immersed in a box of SPC/E water, with a minimum distance of 0.9 nm between protein atoms and the edges of the box. 150 mM of NaCl were then added using genion. Long range electrostatics were treated with the particle-mesh Ewald summation^86^. Bond lengths were constrained using the P-LINCS algorithm. The integration time step was 5 fs. The v-rescale thermostat and the Parrinello–Rahman barostat were used to maintain a temperature of 300 K and a pressure of 1 atm. Each system was energy minimized using 1,000 steps of steepest descent and equilibrated for 500 ps with restrained protein heavy atoms prior to production simulations. Snapshots were extracted every 200 ps from each trajectory, leading to the generation of ~3300 additional models of P_135–237_.

In order to generate models of P_135–294_ and of P_1–294_, we adopted a similar strategy in which residues 238–294 and residues 1–134 were grafted onto the existing P_135–237_ models with or without the α-helical secondary structure elements (residues 14–26 and residues 251–262). Model types that were not selected through ensemble optimization of P_135–237_ were not considered for this procedure. We then used the SBM approach to generate ensembles for P_135–294_ and P_1–294_, yielding ~5000 P_135–294_ and ~8000 P_1–294_ models.

### Ensemble optimization

For each model from each ensemble, theoretical SAXS patterns were calculated with the program CRYSOL^87^ and ensemble optimization fitting was performed with GAJOE^88,89^. GAJOE uses a genetic algorithm to select from a large pool of conformers optimized sub-ensembles that minimize the discrepancy between the experimental and calculated curves *χ*_exp_ according to the following equation:

1 $$\chi {exp}^{2}=\frac{1}{K\,-\,1}\sum _{j=1}^{K}{[\frac{\mu I({Q}_{j})-{I}_{exp}({Q}_{j})}{\sigma ({Q}_{j})}]}^{2}$$

where *K* is the number of points in the experimental curve, σ is the standard deviation and µ is a scaling factor. The optimum selected ensemble size and relative weights of the models were determined automatically by GAJOE. For each curve, the ensemble optimization procedure was repeated for a minimum of 20 times, from which the Rg distributions of the optimized ensembles were built.

### Crystallization and data collection

Our initial goal was the crystallization of a complex of the HMPV M2-1 protein bound to a P construct including the putative M2-1 binding region. The expression and purification of HMPV M2-1 has been described previously^30^. In an attempt to crystallize the M2-1 – P_135–237_ complex, vapour diffusion crystallization trials of a 1:1 mixture of these two proteins at 7 mg/ml in 20 mM Tris, pH 7.5, 150 mM NaCl were set up using a Cartesian Technologies pipetting system^90^. Although we were not able to grow crystals of a complex, crystals which later proved to harbour only the P oligomerization region could be obtained after extended time periods. The P2_1_ crystal (form 1) of HMPV P_core_ grew at 20 °C after 291–344 days with mother liquor containing 20% polyethylene glycol (PEG) 6000, 200 mM NaCl, and 100 mM Tris, pH 8.0. The P2_1_2_1_2_1_ crystal (form 2) grew at 20 °C after between 132 and 185 days with mother liquor containing 25% PEG 3350, 200 mM MgCl, and 100 mM Tris, pH 8.5. Crystals were frozen in liquid nitrogen after being cryoprotected with 25% glycerol. Diffraction data were recorded on beamlines I03 (P2_1_ crystal) and I04 (P2_1_2_1_2_1_ crystal) at Diamond Light Source, Didcot, UK. Data reduction was carried out automatically with XIA2^91^.

### Structure determination and refinement

The HMPV P_core_ data sets were phased by molecular replacement with PHASER^92^ using the previously published structure (pdbID:4BXT). The structures from both crystal forms were subjected to multiple rounds of manual building in COOT^93^ and refinement in PHENIX^94^. We made use of translation-libration-screw (TLS) parameters and 8-fold torsion-angle non-crystallographic symmetry (NCS) restraints as implemented in PHENIX^94^. For the 1.6 Å data of crystal form 1 we additionally carried out anisotropic atomic displacement parameter (ADP) refinement. The structures were validated with the wwPDB Validation Service (https://validate-rcsb-1.wwpdb.org/). Refinement statistics are given in Table 1. The final coordinates and structure factors have been deposited in the PDB with accession codes 5OIX and 5OIY.

### Structure and sequence analyses

Structure-related figures were prepared with the PyMOL Molecular Graphics System (DeLano Scientific LLC). Protein interfaces were analysed with the PISA webserver^95^. Mapping of sequence conservation onto the P_core_ structure was carried out with the ConSurf server^96^ using P sequences from the *Pneumoviridae* family members human metapneumovirus (HMPV), avian metapneumovirus (AMPV), canine pneumonia virus (CPV), murine pneumonia virus (MPV), bovine respiratory syncytial virus (BRSV), and human respiratory syncytial virus (HRSV). Sequences were aligned using using PROMALS3D^97^ and Jalview^98^ in order to analyse the conservation of the protein binding sites that were identified in RSV. The putative functional regions have been assigned based on homology and previously reported studies^16,26,27,42,60,66,99,100^. The linear net charge per residue (NCPR) for HMPV P was calculated using the Classification of Intrinsically Disordered Ensemble Regions (CIDER) webserver^101^.

### Data availability

Coordinates and structure factors have been deposited in the Protein Data Bank with accession numbers 5OIX and 5OIY. The SAXS datasets generated during the current study are available from the corresponding author on reasonable request.

## Electronic supplementary material

Supplementary Figure S1–5
