# Supplementary material for: Structural dissection of human metapneumovirus phosphoprotein using small angle x-ray scattering
Source: Sci Rep. 2017 Nov 1;7:14865. doi: 10.1038/s41598-017-14448-z (PMC5665942; doi:10.1038/s41598-017-14448-z)
Supplement: Supplementary file 1 — Supplementary Figure S1–5 [file 41598_2017_14448_MOESM1_ESM.pdf]

SUPPLEMENTARY INFORMATION

Structural dissection of human metapneumovirus phosphoprotein using small angle x-ray scattering

Max Renner[1], Guido C. Paesen[1], Claire M. Grison[2], Sébastien Granier[2], Jonathan M. Grimes*[1,3] and Cédric Leyrat*[2]

[1]Division of Structural Biology, Wellcome Trust Centre for Human Genetics, Oxford University, Roosevelt Drive, Oxford, OX3 7BN, UK

[2]Institut de Génomique Fonctionnelle, CNRS UMR-5203 INSERM U1191, University of Montpellier, Montpellier, France.

[3]Science Division, Diamond Light Source Ltd., Diamond House, Harwell Science and Innovation Campus, Didcot, Oxfordshire, OX11 0DE, United Kingdom

*To whom correspondence should be addressed: jonathan@strubi.ox.ac.uk or cedric.leyrat@igf.cnrs.fr

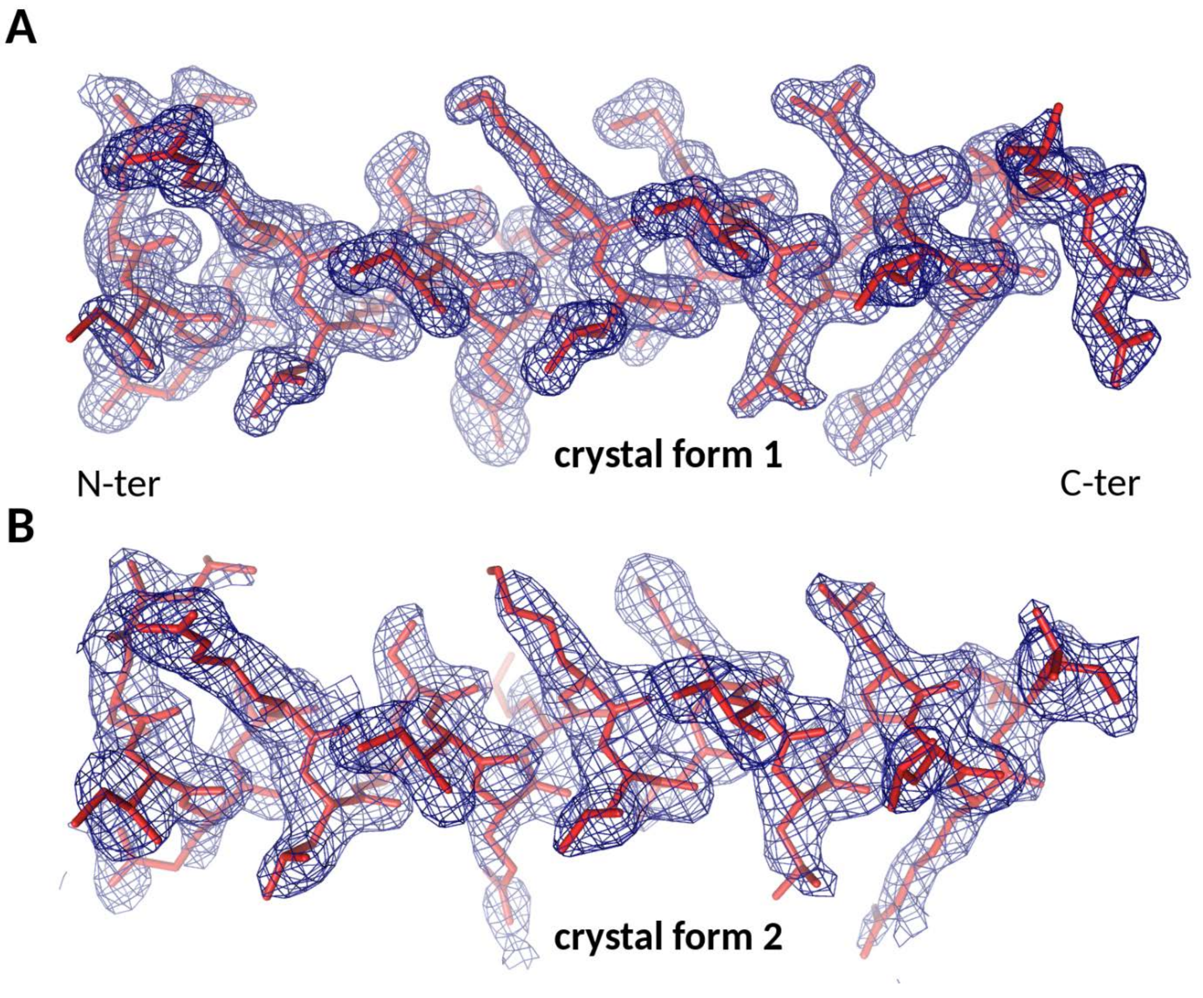

Figure S1: Electron density maps of P_{core} crytals. A, B. Sample 2Fo-Fc maps contoured at 1.0 σ for one helix each of the 4-helix bundles in the new crystal forms of P_{core}. N- and C-termini are labelled. The fitted models are shown in red in stick representation.

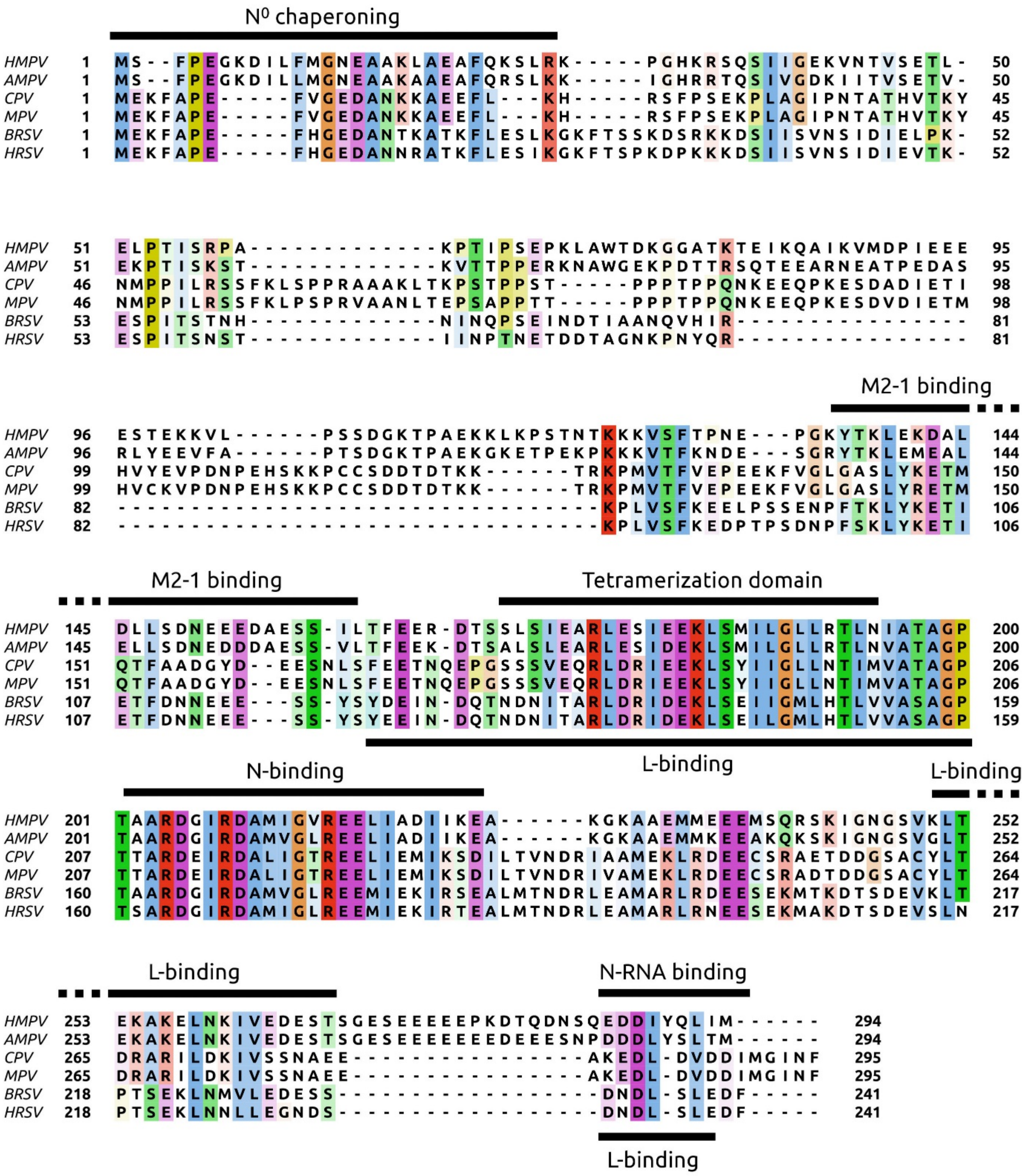

Figure S2: Sequence alignment of *Pneumoviridae* P proteins with approximate and putative annotation of functional regions. The multiple sequence alignment was generated using PROMALS3D [97] and Jalview [98]. Residues are colored using the ClustalX scheme, as implemented in Jalview. The putative functional regions have been assigned based on homology and previously reported studies [16, 26, 27, 42, 60, 66, 99, 100] . HMPV, human metapneumovirus, AMPV, avian metapneumovirus, CPV, canine pneumonia virus, MPV, murine pneumonia virus, BRSV, bovine respiratory syncytial virus, HRSV, human respiratory syncytial virus.

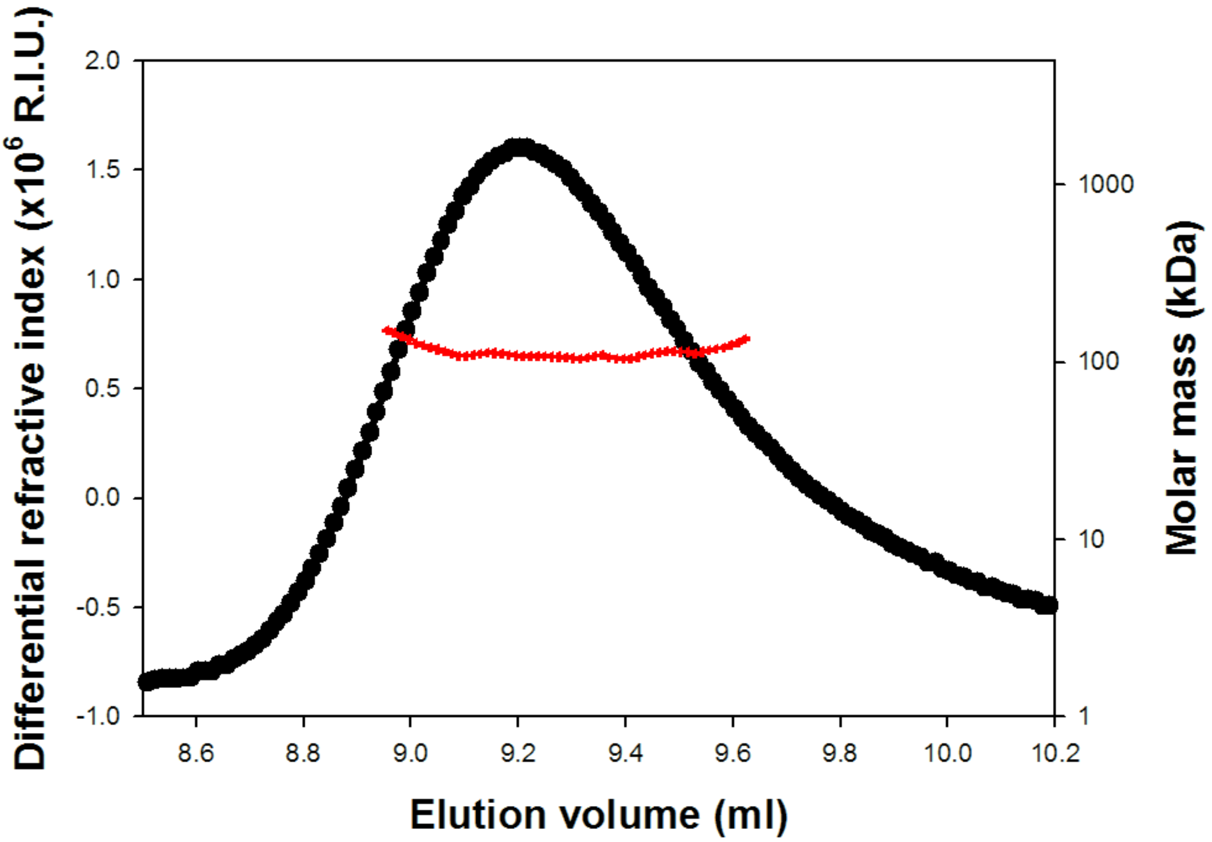

Figure S3: Characterization of full-length HMPV P by size exclusion chromatography (SEC) combined with detection by multi-angle laser light scattering (MALLS) and refractometry (RI). The black line shows the SEC elution profile as monitored by refractometry. The red line shows the molecular weight calculated from light scattering and refractometry data.

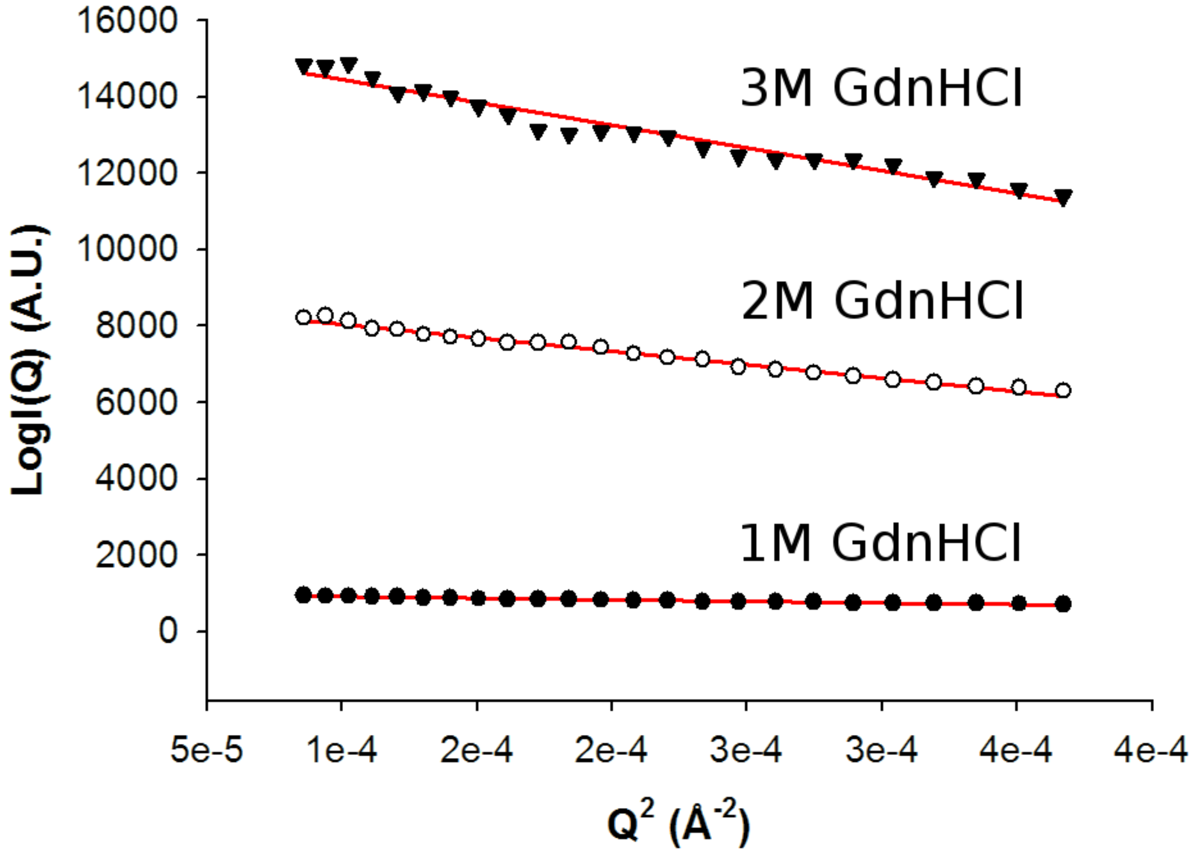

Figure S4: Guinier plots for P$_{135\text{-}237}$ SAXS profiles measured in the presence of guanidinum hydrochloride, showing linear behavior in the low Q range.

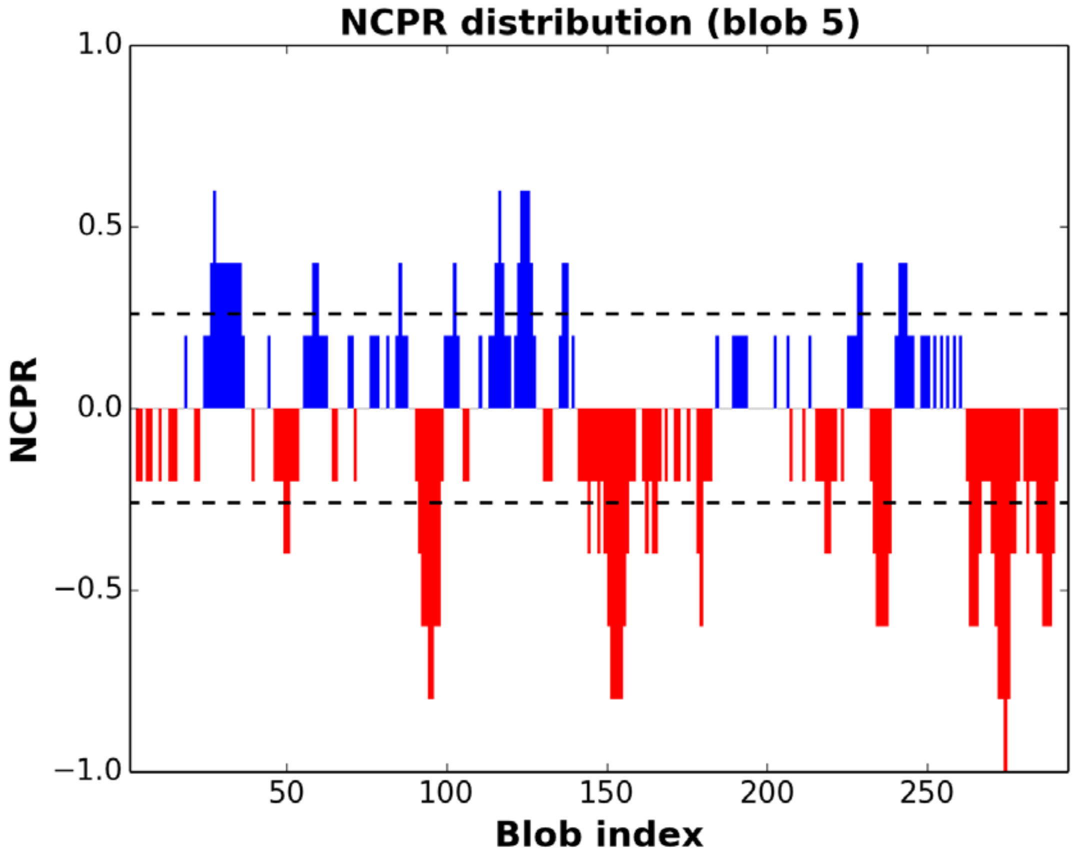

Figure S5: Linear net charge per residue (NCPR) for HMPV P. Plot of NCPR over 5 residue windows for the HMPV P sequence. The plot was obtained using the Classification of Intrinsically Disordered Ensemble Regions (CIDER) webserver [101].
